# Genetic Background of Blood β-Hydroxybutyrate Acid Concentrations in Early-Lactating Holstein Dairy Cows Based on Genome-Wide Association Analyses

**DOI:** 10.3390/genes15040412

**Published:** 2024-03-26

**Authors:** Yueqiang Wang, Zhenyu Wang, Wenhui Liu, Shuoqi Xie, Xiaoli Ren, Lei Yan, Dong Liang, Tengyun Gao, Tong Fu, Zhen Zhang, Hetian Huang

**Affiliations:** 1 College of Animal Science and Technology, Henan Agricultural University, Zhengzhou 450046, China; wzyhan2017@163.com (Z.W.); lwh0285@163.com (W.L.); xsq990825@163.com (S.X.); wangyueqiang1985@126.com (Y.W.); liangdong12388@foxmail.com (D.L.); dairyfarm@163.com (T.G.); futong2004@126.com (T.F.); 2 College of Animal Science, Anhui Science and Technology University, Fengyang 233100, China; 3 Henan International Joint Laboratory of Nutrition Regulation and Ecological Raising of Domestic Animal, Zhengzhou 450046, China; 4 Henan Dairy Herd Improvement Center, Zhengzhou 450046, China; renxl1990@163.com (X.R.); yanleihcy@163.com (L.Y.)

**Keywords:** ketosis, estimate genetic parameters, GWAS, Chinese Holstein cattle

## Abstract

**Simple Summary:**

Ketosis (KET), a metabolic disorder frequently observed in dairy cows, is characterized by increased levels of ketone bodies. The “gold standard” for diagnosing ketosis is the concentration of blood β-hydroxybutyrate (BHB). The increasing number of studies focusing on BHB highlights the increasing significance of metabolic disorders in both the dairy industry and the scientific community. Additionally, the surge in research on the genetic and economic facets of the KET is likely correlated with the recent accessibility of extensive datasets containing blood BHB concentrations as routinely recorded traits in certain production systems. Such data are essential to accurately estimating the genetic parameters associated with these traits. Consequently, the objective of this study was to estimate genetic parameters and identify candidate genes related to blood BHB through genome-wide association analysis to provide research directions for dairy ketosis.

**Abstract:**

Ketosis is a common metabolic disorder in the early lactation of dairy cows. It is typically diagnosed by measuring the concentration of β-hydroxybutyrate (BHB) in the blood. This study aimed to estimate the genetic parameters of blood BHB and conducted a genome-wide association study (GWAS) based on the estimated breeding value. Phenotypic data were collected from December 2019 to August 2023, comprising blood BHB concentrations in 45,617 Holstein cows during the three weeks post-calving across seven dairy farms. Genotypic data were obtained using the Neogen Geneseek Genomic Profiler (GGP) Bovine 100 K SNP Chip and GGP Bovine SNP50 v3 (Illumina Inc., San Diego, CA, USA) for genotyping. The estimated heritability and repeatability values for blood BHB levels were 0.167 and 0.175, respectively. The GWAS result detected a total of ten genome-wide significant associations with blood BHB. Significant SNPs were distributed in *Bos taurus autosomes* (BTA) 2, 6, 9, 11, 13, and 23, with 48 annotated candidate genes. These potential genes included those associated with insulin regulation, such as *INSIG2,* and those linked to fatty acid metabolism, such as *HADHB*, *HADHA*, and *PANK2*. Enrichment analysis of the candidate genes for blood BHB revealed the molecular functions and biological processes involved in fatty acid and lipid metabolism in dairy cattle. The identification of novel genomic regions in this study contributes to the characterization of key genes and pathways that elucidate susceptibility to ketosis in dairy cattle.

## 1. Introduction

Ketosis is a common metabolic disorder that affects dairy cows in the early postpartum period, typically detected by analyzing blood β-hydroxybutyrate levels. The incidence of subclinical ketosis among Holstein cows across ten countries varies from 11.2% to 36.6%, with an average of 21.8% [1]. The onset of ketosis results in increased costs for dairy farms, including expenses related to ketosis treatment, an increased risk of other diseases, compromised reproductive performance, and the likelihood of early lactation culling [2,3,4]. Therefore, mitigating the incidence of ketosis improves the health of dairy cows as well as reduces production costs on dairy farms.

Dairy cows exhibit individual differences in metabolic adaptability during the early lactation period, which can be leveraged to select high-yielding cows with a reduced risk of metabolic disorders [5]. Previous genetic studies of ketosis, predominantly relying on clinical records, have revealed heritability estimates ranging from 0.01 to 0.16, indicating a low heritability level [6]. The low heritability of ketosis suggests that achieving significant genetic progress through direct genetic selection methods is difficult. Blood BHB is the predominant and most stable ketone body in bodily fluids. Its concentration is commonly employed as a “gold standard” for ketosis and provides a more accurate indication of sensitivity to ketosis than other ketone bodies [7,8,9]. In addition, blood BHB exhibits moderate heritability (0.17 to 0.40) [10,11,12,13,14]. Genetic enhancement and genomic selection strategies with BHB as a phenotype may effectively reduce the occurrence of ketosis. It is crucial to meticulously record specific traits like ketone body concentrations (acetone or BHB) in blood or milk to detect ketosis (KET). Therefore, employing BHB as an indicator trait for indirect selection in ketosis should yield greater genetic gains than direct selection.

Rapid advancements in sequencing technology have revealed causal variants of complex traits through genome-wide association analysis [10,11,12]. Several studies have investigated the physiological, quantitative genetic, and genomic associations related to milk BHB, acetone, and KET. Huang et al. [13] identified six genomic regions associated with ketosis based on the binary ketosis trait in production records. Freebern et al. [14] conducted a GWAS and fine-mapping analysis to identify potential candidate genes associated with disease traits in Holstein cattle. They identified one significant segment that encompassed DGAT1 on BTA14 for KET. Pralle et al. [15] performed a GWAS based on a KET phenotype determined by repeated blood BHB concentrations and identified several novel marker associations and metabolic pathways contributing to the genetic risk of dairy ketosis. However, previous studies have not directly reported significant genome-wide regions or genes containing putative causative mutations associated with blood BHB concentrations. Therefore, this study aimed to investigate the genetic parameters and genomic associations associated with blood BHB concentrations in early lactating Holstein cows in China. The purposes of this study were to (1) estimate (co)variance components based on pedigree and genomic data, (2) conduct a GWAS to identify significantly associated SNPs, and (3) annotate and provide biological interpretations of potential candidate genes.

## 2. Materials and Methods

### 2.1. Ethics Statement

All animals were treated in accordance with the protocols approved by the Institutional Animal Care and Use Committee of Henan Agriculture University (Permit Number: 12-1328).

### 2.2. Phenotype

Blood BHB concentrations were measured in 45,617 Holstein cows during the first three weeks after calving, from December 2019 to August 2023. Data were gathered from seven dairy-intensive pasture farms located in northern China (Table 1). The free-stall barn system and TMR were implemented in every herd, with an average of 6517 ± 2751 cows and 11,982 ± 5219 records per herd. Blood BHB concentration data were obtained from production records at the dairy farm. Within the first three weeks postpartum in Holstein cows, veterinarians collected blood samples from the tail vein. The blood samples were tested for BHB concentration using a handheld FreeStyle Optium Neo H ketone Meter (Abbott Diabetes Care Ltd., Witney, UK). The device had a sensitivity of 98% and a specificity of 95% [16].

A dataset containing records of cows with days in milk (DIM) from days 1 to 21 and blood BHB values ranging from 0.1 to 8.0 mmol/L was kept for analyses. The dataset included a total of 83,878 blood BHB records from 45,617 Holstein cows. BHB concentrations in cows were categorized based on parity, with cows in their first, second, and third or greater parities labeled as BHB1, BHB2, and BHB3, respectively. The number of animals in each category was 27,261, 30,933, and 25,684, respectively. Furthermore, pedigrees can be traced back three generations.

### 2.3. Genotype and Quality Control

Blood samples were collected from the coccygeal vein and transferred into 10 mL vacuum tubes containing EDTAK2 as an anticoagulant. The samples were promptly cooled on ice to preserve their integrity. Genomic DNA was subsequently extracted from the blood samples of 5146 Holstein dairy cows using the phenol chloroform method. Most of these animals (*n* = 3919) were genotyped with the Geneseek Genomic Profiler (GGP) Bovine 100 K SNP Chip by Neogen Biotechnology (Shanghai, China), and a minority of these animals (*n* = 1227) were genotyped using the GGP Bovine SNP50 v3 (Illumina Inc., San Diego, CA, USA). Using the 100 K chip reference data of 3919 Holstein dairy cows, the 50 K chip data of 1227 Holstein dairy cows were imputed to the 100 K chip data with an average imputation accuracy (R^2^) of 0.981 and ARS-UCD1.2(bosTau9) was used as a reference genome. Genotype imputation was performed using Beagle software (version 5.4) [17]. Meanwhile, quality control (QC) was conducted using PLINK 1.90 [18] to remove the SNPs that did not meet the specific criteria: (1) the individual genotype call rate < 95%, (2) the SNP genotype call rate < 90%, and (3) the minor allele frequency (MAF) < 0.01 and deviated from the Hardy–Weinberg equilibrium value (*p* < 1.0 × 10^−6^). After quality control, 5139 cows and 80,217 SNP variants were used for further association analyses.

### 2.4. Estimation of Genetic Parameters

Generalized linear models were performed using the GLM function [19] implemented in the Basic package of R 4.3.2 [20], with blood BHB as the response variable to identify the systematic effects that should be included in the genetic models. A single-trait repeatability animal model (model 1) was used to estimate the heritability and repeatability of blood BHB. The model 1 can be written as follows:

(1) $$y_{ijklmn}={HYS}_{i}+P_{j}+{DIM}_{k}+a_{l}+{pe}_{m}+\varepsilon_{ijklmn},$$

where $y_{ijklmn}$ are the phenotypic records for blood BHB; ${HYS}_{i}$ is the fixed effect of the herd-year-season i (104 levels); $P_{j}$ is the fixed effect of parity j (1, 2, or 3+); ${DIM}_{k}$ is the covariate effect; $a_{l}$ is the random additive genetic effect; ${pe}_{m}$ is the random permanent environmental effect; and $\varepsilon_{ijklmn}$ is the random residual effect. Two different methods were employed to estimate variance components and predict breeding values: (a) the pedigree-based approach, which employed the BLUP method. In this method, the covariance structure of the random animal effect was modeled as u ∼ N(0,A$\sigma_{a}^{2}$), where A represents the pedigree relationship matrix and $\sigma_{a}^{2}$ denotes the direct additive genetic variance, and genetic parameters were analyzed using the AIREMLF90 program [21,22]; (b) The genomic evaluation implemented a single-step genomic BLUP (ssGBLUP) method, which modeled the random animal effect as u ∼ N(0,H$\sigma_{a}^{2}$), with H being a matrix that integrates pedigree and genomic relationships [23].

### 2.5. Genome-Wide Association Study

We employed the FarmCPU method, implemented using rMVP [24] in R version 4.3.2 for GWAS in this study. The FarmCPU method utilizes the fixed-effect model (FEM) and the random-effect model (REM) iteratively [25]. The FEM is applied to test each of the m genetic markers individually. Pseudo-quantitative trait nucleotides (QTNs) are included as covariates to control false positives. Specifically, the FEM can be described as follows:

(2) $$Y_{i}=M_{i1}b_{1}+M_{i2}b_{2}+M_{i3}b_{3}+\cdots +M_{it}b_{t}+S_{ij}d_{j}+e_{i},$$

where $Y_{i}$ is the observation on the *i*th sample; $M_{i1},M_{i2},M_{i3}$⋯$M_{it}$ represents the genotypes of the t pseudo-QTNs; $b_{1},b_{2},b_{3},\cdots$,$b_{t}$ is equal to the corresponding effect for the pseudo QTNs; $M_{ij}$ represents the genotype of the jth SNPs and ith sample; $S_{j}$ represents the corresponding effect of the jth SNPs; and $e_{i}$ represents the residual.

The REM is employed to optimize the selection of pseudo QTNs from markers based on their testing statistics and positions using the SUPER algorithm [26] in an REM, as below:

(3) $$Y_{i}=U_{i}+e_{i},$$

where $Y_{i}$ is the observation of the ith sample, the BHB concentration and estimated breeding values (EBVs) of BHB were used as the phenotype, respectively; $e_{i}$ is the residual, and $U_{i}$ represents the total genetic effect of the ith sample. We determined the threshold value for selecting significant SNPs using the Bonferroni correction method [27]. The genome-wide significant level was set at 6.23 × 10^−7^ (0.05/80,217).

### 2.6. Functional Analyses

Gene set enrichment analysis helps identify phenotype-relevant SNPs and mechanisms of action by identifying corresponding biological pathways. Genes within 500 kb of the identified SNPs were used for further functional analyses. BioMart from Ensembl (release 101) was used to extract gene names and descriptions using the bovine genome assembly ARS-UCD1.2. Subsequently, we verified the biological functions of the relevant genes using the NCBI GenBank database (https://www.ncbi.nlm.nih.gov/, accessed on 10 December 2023) and carefully selected genes associated with the study. The online Gene Ontology (GO) database and Kyoto Encyclopedia of Genes and Genomes (KEGG) database were searched using the KOBAS [28] (http://bioinfo.org/kobas, accessed on 22 December 2023) according to the protein-coding gene ID. Statistical significance was set at *p* < 0.05. Finally, gene networks were constructed based on the predicted protein interactions among the annotated genes, using the STRING database (www.string-db.org/, accessed on 30 December 2023) [29].

## 3. Results

### 3.1. Descriptive Statistics

The descriptive statistical results of seven cattle herds are shown in Table 2. The blood ketone levels ranged from 0.1 to 8.0 mmol/L, with a mean of 0.927 mmol/L and a standard deviation of 0.527 mmol/L. Specifically, the highest average value was observed for H2 at 1.01 mmol/L, whereas the lowest average value was observed for H1 at 0.868 mmol/L. The number of animals with genotype and blood BHB phenotype data for different parities is presented in Supplementary Table S1.

### 3.2. Estimation of Genetic Parameters

The genetic parameters estimated from the pedigree-based and genomics-based analyses are listed in Table 3. BHB heritability in the present study ranged from 0.167 to 0.169, depending on the different information analyses used. In general, $h^{2}$ estimates with genomic information were slightly higher than those with pedigree information, mainly because of the larger additive genetic variance (Table 3). Considering that the residual variance was almost the same in the two approaches, the inclusion of genomic information resulted in the same variance, from the permanent environmental variance to the additive genetic effect.

### 3.3. Genome-Wide Association Study

**Figure 1:**
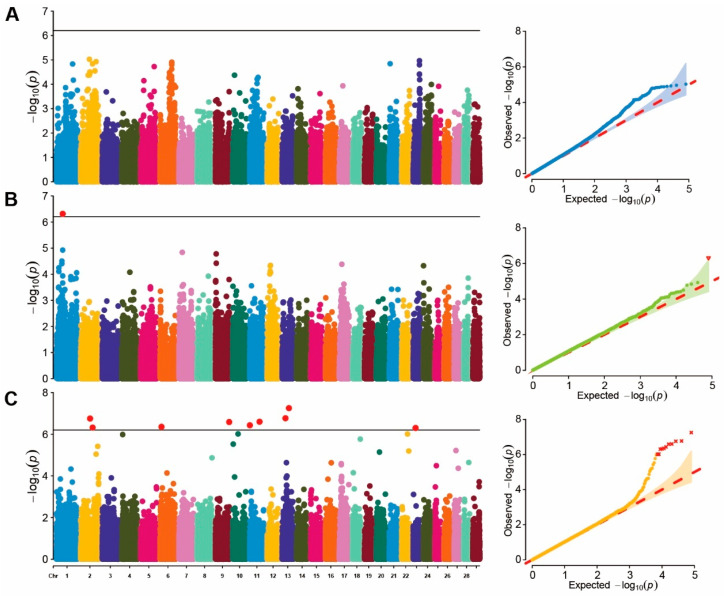
Manhattan and QQ plot of GWAS. (**A**) represents the Manhattan and QQ plots for the second-parity GWAS; (**B**) represents the Manhattan and QQ plots for the third-parity GWAS; (**C**) represents the Manhattan and QQ plots for the breeding values GWAS.

A single-marker GWAS analysis was conducted on blood BHB values in cows of different parities. Due to limited chip data for first-parity cows, genome-wide association analysis was performed on data from second- and third-parity cows. The *p*-value profiles of all SNP markers associated with each parity group are represented in Figure 1. No significant SNP loci were found in the second-parity GWAS, whereas only one significant SNP locus was identified in the third-parity GWAS. This SNP locus, located at ARS-BFGL-NGS-41577 in BTA 1 (position: 46.75 Mb), was annotated as *ZPLD1*. In addition, the effect of each SNP was calculated using the FarmCPU method based on the EBVs of all parity groups. (Figure 1C). The results revealed that nine significant SNP were distributed across BTA 2, 6, 9, 11, 13, and 23 (Table 4), among which the region on BTA 13 (50,955,987 bp) was the most significant loci (${-log}_{10}P>6.20$). A total of 48 genes located within 500 Kb of the significant SNPs were identified as potential candidate genes for the investigated traits.

### 3.4. Functional Analyses

**Figure 2:**
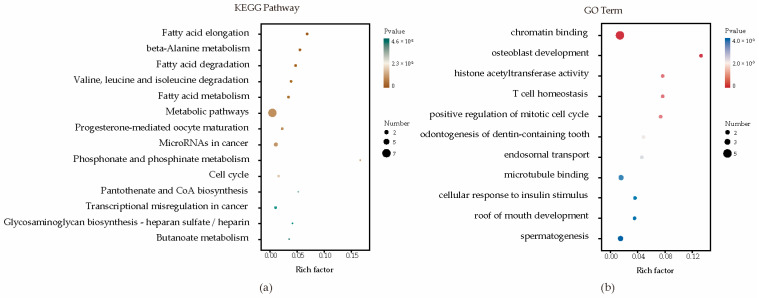
Enrichment analysis factor diagram. (**a**) Enrichment analysis of KEGG metabolic pathways; (**b**) Enrichment analysis of GO metabolic pathways.

**Figure 3:**
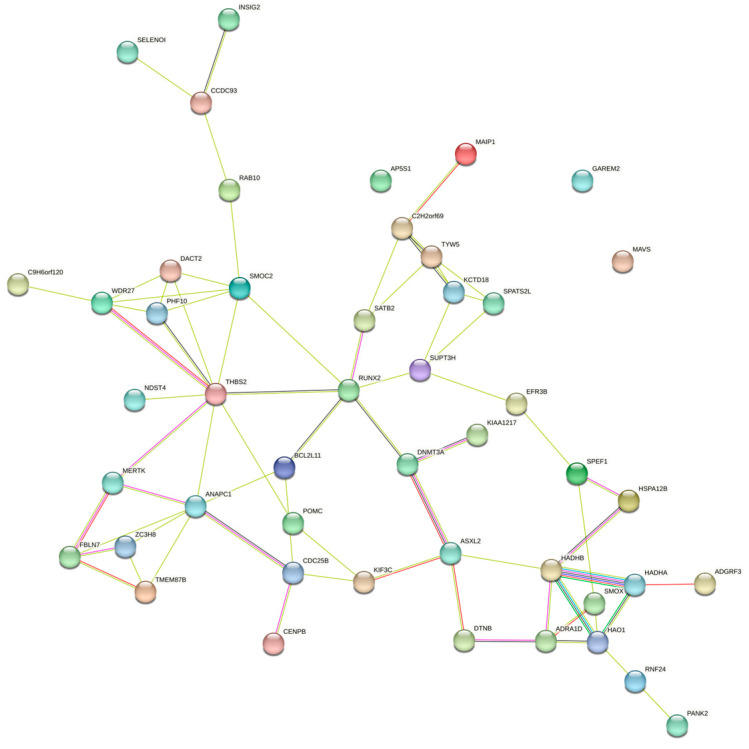
Gene interaction network for the central genes associated with blood BHB.

Enrichment analyses for GO terms and KEGG pathways were conducted on all candidate genes to pinpoint their roles in established metabolic pathways. Based on functional annotation results, significant pathways were identified for blood BHB (Figure 2; Supplementary Table S2). These comprised various KEGG pathways, including fatty acid elongation, β-alanine metabolism, fatty acid degradation, Valine, leucine, and isoleucine degradation, and fatty acid metabolism. Furthermore, they comprised GO terms such as chromatin binding, osteoblast development, histone acetyltransferase activity, T cell homeostasis, and positive regulation of the mitotic cell cycle. The relationships among the genes in the network were established using various methods, such as co-expression, gene fusion, protein homology, gene neighborhood, and gene co-occurrence. A network comprising 44 nodes connected by 65 edges was constructed (Figure 3).

## 4. Discussion

The present study aimed to estimate the genetic parameters of blood BHB and discover genomic variants associated with blood BHB using GWAS. In a large dataset of early lactating Holstein cows, most heritability estimates for BHB were based on predicted results from milk infrared spectra. For example, Koeck et al. [30] reported $h^{2}$ values ranging from 0.14 to 0.29 for Canadian Holsteins. Similarly, comparable heritability values were observed for Korean Holsteins, ranging from 0.14 to 0.09 in the first and fourth parities, respectively [5]. In Italian Holsteins, blood BHB had a moderate heritability in the first 35 days of milk, ranging from 0.13 to 0.3 [31]. The heritability in the present study was higher than previous estimates based on blood BHB predicted from the milk infrared spectra of Chinese Holstein cattle, in which the BHB heritability estimates ranged from 0.100 to 0.131 [32]. Continuous phenotypes, such as blood BHB concentrations, appear to serve as superior indicators of ketosis susceptibility for implementation in breeding programs. In a previous study, the estimated heritability of blood BHB concentration in primiparous Holstein cows, using a random regression model, ranged from 0.08 to 0.40 [33], which were significantly different from our results, mainly because they focused on primiparous cows and their sample size was smaller. The disparities noted in the aforementioned studies are likely attributable to variations in population, dataset structure, and statistical models employed. The results of another study were similar to those of the present study. Drift et al. [9] conducted a genetic analysis using early lactation blood and milk BHB in Dutch Holstein cows, revealing a heritability of 0.17 for blood BHB. Overall, the heritability estimates of blood BHB levels were consistent with those reported in previous studies.

Furthermore, using the BLUP and ssGBLUP analyses, the variance components and heritability estimates for blood BHB were 0.167 and 0.169, respectively (Table 3). The ssGBLUP method, which integrates genomic and pedigree relationship matrices, enhances the accuracy of breeding values for production traits in young animals compared to conventional BLUP methods [23,34,35]. The incorporation of genomic data enhances the precision of kinship assessments, thereby facilitating more precise estimations of genetic relationships among individuals. Our findings indicated a marginal increase in heritability estimates when using genomic data compared to pedigree-based methodologies. The limited increase in heritability may be attributed to the inclusion of individuals in the pedigree data. The inclusion of genomic data substantially improves the reliability of these traits [36,37]. In the future, we plan to collect more samples during periods with the highest phenotypic and genetic variation.

In our genome-wide association study, we performed a single-marker GWAS analysis using blood ketone values across different parity groups. However, no significantly associated SNP was found during the second lactation period, and all SNP effects were too small to be significant when the Bonferroni correction was applied. Only one SNP was significantly correlated with blood BHB levels during the third lactation period. It is important to recognize that the efficacy of GWAS is influenced by variables such as sample size and the density of SNP loci. Detecting SNPs with small effects can be difficult in circumstances of limited sample sizes or sparse SNP marker coverage [38,39]. Notably, we identified nine significant SNP loci located on BTA 2, 6, 9, 11, 13, and 23 based on BHB breeding values. The SNP BTA-32905-no-rs (50,955,987 bp) on BTA 13, which exhibited the highest −log10 *p*-value of 5.57 × 10^−7^, was situated within the genes *RNF24*, *PANK2*, and *MAVS*. However, further research is needed to explore the relationship between these three candidate genes and ketosis. On BTA 11, the two significant SNP loci (BovineHD1100000220, 730,613 bp, and BovineHD1100021063, 73,677,409 bp) included the potential candidate genes *HADHA*, *HADHB*, *BCL2L11*, and *EFR3B*. In particular, the gene hydroxyacyl-CoA dehydrogenase trifunctional multienzyme complex subunit α (*HADHA*) and β (*HADHB*) within the defined interval surrounding SNP BovineHD1100021063 (at 73,677,409 bp) on BTA 11 are associated with subclinical ketosis in later lactation [40]. In addition, studies have suggested the involvement of the *BCL2L11* gene in lipid and glucose metabolic pathways [41]. The *EFR3B* gene is considered a candidate gene for human type 1 diabetes [42]. On BTA 2, both the SNP BovineHD0200020100 (69,386,940 bp) and BovineHD0200025237 (88,536,618 bp) exceeded the significance threshold. One of the candidate genes encompassing these two SNPs was *INSIG2*. The insulin-regulating gene *INSIG2* is involved in insulin signal transduction and plays a crucial role in the regulation of lipid synthesis in mammary epithelial cells [43,44]. Insulin is a vital metabolic hormone that plays a key role in the regulation of energy metabolism during the transition period in dairy cows [45]. In the pathogenesis of ketosis, insulin resistance has been proposed as a predisposing factor because it may facilitate excessive postpartum adipose triglyceride lipolysis [46,47]. A previous GWAS of KET also detected several candidate genes associated with insulin metabolism and insulin-dependent diabetes [48]. Therefore, the *INSIG2* gene may be considered a new candidate gene for KET. In addition to these findings, we detected significant SNP loci on BTA 6, 9, and 23 and annotated them to certain candidate genes.

To further annotate the functions, enrichment analysis was conducted on a list of candidate genes. These results indicated that molecular functions and biological processes are associated with fatty acid, amino acid, and lipid metabolism in dairy cattle. These comprise various KEGG pathways, including bta00062 (fatty acid elongation), bta00410 (β-alanine metabolism), bta00071 (fatty acid degradation), bta00280 (Valine, leucine, and isoleucine degradation), and bta01212 (fatty acid metabolism). The regulation of fatty acid metabolism directly influences the energy balance in dairy cows. High-yielding cows often experience ketosis more easily because of their higher demand for glucose and energy, which can lead to an insufficient glucose supply, prompting the breakdown of fatty acids and ketones [49,50]. Moreover, the gene–gene network analysis proved highly effective in exploring shared biological processes among candidate genes. *HADHA* and *HADHB* together form a mitochondrial membrane-bound hetero-oligomeric complex that catalyzes the final three steps of mitochondrial long-chain fatty acid β-oxidation. In a study on the glucagon response in mouse liver, under conditions of glucose scarcity, the mitochondrial β-oxidation enzyme *HADHA* promoted the production of β-hydroxybutyrate through β-oxidation [51]. In another study on liver tissue across three stages of lactation in Holstein cows, *HADHB* was identified as a candidate gene for milk fat, casein, and lactose synthesis in dairy cows [52]. Overall, these insights into the potential involvement of candidate genes contributing to susceptibility to ketosis may offer a foundational framework for understanding the pathogenesis of ketosis.

The results from our study confirmed the polygenic background of blood BHB and KET, indicating their influence on numerous genomic regions, likely with small effects. One limitation of the current study is the small sample size of cows included in the GWAS analysis, which, when combined with the statistical adjustment for multiple testing, may lead to a reduced number of significant SNPs being detected [53]. All SNP effects were too small to reach significance when considering the strict Bonferroni-corrected *P*-value. McCarthy et al. [54] suggested increasing the sample size to enhance the statistical power in GWAS for complex traits with low incidences. Additionally, genotyping cows with a denser SNP chip, as demonstrated by Freebern et al. [14], could potentially influence significance tests in GWAS. In future research, we intend to expand the sample size and utilize high-density SNP chip genotyping to improve the statistical power of GWAS for complex, low-heritability traits. Meanwhile, we will also plan to identify key candidate genes associated with ketosis by integrating genome-wide association and transcriptome data.

## 5. Conclusions

This study identified the genomic regions associated with blood BHB, and the estimated heritability and repeatability values for blood BHB were 0.167 and 0.175, respectively. The loci on BTA 2, 6, 9, 11, 13, and 23 exhibited significant associations with blood BHB values and were considered to harbor crucial genes related to ketosis. These findings uncovered important biological pathways related to fatty acid metabolism and lipid metabolism in dairy cattle. To address the limitations of this study, we intend to increase the sample size in future research and utilize high-density SNP chip genotyping to enhance the statistical power of GWAS for complex, low-heritability traits. In summary, our result provides a promising resource of candidate genes associated with blood BHB and ketosis in cattle, which can be utilized in breeding programs and future investigations into disease genes for clinical applications.

## Figures and Tables

**Table 1:** Summary statistics.

Items	Number
Number of records	83,878
Number of animals	45,617
Number of herd-years-season	104
Number of parities	3
Mean incidence of ketosis (%)	1.1
Number of genotyped individuals	5146
Number of SNP after editing	80,217

**Table 2:** Descriptive statistics of blood BHB trait in Chinese Holstein cattle.

Herd	n *	No. of Cows	Mean	SD	Min	Max
H1	14,691	9134	0.868	0.469	0.1	6.1
H2	16,612	8367	1.010	0.516	0.1	5.4
H3	16,749	8798	0.937	0.507	0.1	8.0
H4	15,614	8107	0.925	0.529	0.1	8.0
H5	11,905	6363	0.888	0.485	0.1	5.4
H6	2855	1570	0.880	0.478	0.1	4.4
H7	5451	3278	0.926	0.785	0.1	7.3

* n = number of records.

**Table 3:** Estimates of genetic parameters and variance components for blood BHB in Chinese Holstein cattle.

Methods	$\sigma_{a}^{2}$ ± SE	$\sigma_{pe}^{2}$ ± SE	$\sigma_{e}^{2}$ ± SE	$h^{2}$ ± SE	$r_{e}$
BLUP	0.044 ± 0.002	0.002 ± 0.003	0.218 ± 0.002	0.167 ± 0.010	0.175
ssGBLUP	0.045 ± 0.003	0.002 ± 0.002	0.218 ± 0.002	0.169 ± 0.010	0.175

$\sigma_{a}^{2}$ = additive genetic variance; $\sigma_{pe}^{2}$ = permanent environmental variance; $\sigma_{e}^{2}$ = residual variance; $r_{e}$ = repeatability; SE = standard errors.

**Table 4:** Significant SNPs identified for blood BHB in Chinese Holstein cattle.

SNP	BTA	Position	*p*-Value	Gene
BovineHD0200020100	2	69,386,940	1.76 × 10^−7^	*CCDC93, INSIG2*
BovineHD0200025237	2	88,536,618	4.77 × 10^−7^	*SATB2, C2H2orf69, TYW5, MAIP1, SPATS2L, KCTD18*
BTA-05080-no-rs	6	10,139,671	4.39 × 10^−7^	*NDST4*
Hapmap51347-BTA-90657	9	103,006,480	2.59 × 10^−7^	*DACT2, SMOC2, THBS2, WDR27, C9H6orf120, PHF10*
BovineHD1100000220	11	730,613	3.70 × 10^−7^	*ZC3H8, FBLN7, TMEM87B, MERTK, ANAPC1, BCL2L11*
BovineHD1100021063	11	73,677,409	2.51 × 10^−7^	*SELENOI, ADGRF3, HADHB, HADHA, GAREM2, RAB10, KIF3C, ASXL2, DTNB, DNMT3A, POMC, EFR3B*
Hapmap43234-BTA-31907	13	24,940,514	1.70 × 10^−7^	*KIAA1217*
BTA-32905-no-rs	13	50,955,987	5.57 × 10^−7^	*HAO1, ADRA1D, SMOX, RNF24, PANK2, MAVS, AP5S1, CDC25B, CENPB, SPEF1, ADISSP, HSPA12B*
BovineHD2300004717	23	18,540,309	4.98 × 10^−7^	*SUPT3H, RUNX2*

## Data Availability

All data generated or used during this study are available upon request from the corresponding author.

## Supplementary Materials

The following supporting information can be downloaded at https://www.mdpi.com/article/10.3390/genes15040412/s1, Table S1: Descriptive statistics of blood BHB trait for individuals with genotypes in different parity; Table S2: The Kyoto Encyclopedia of Genes and Genomes (KEGG) pathways and Gene Ontology (GO)involved in candidate genes (*p* < 0.05).

## References

[1] Suthar V.S., Canelas-Raposo J., Deniz A., Heuwieser W. (2013). Prevalence of subclinical ketosis and relationships with postpartum diseases in European dairy cows. J. Dairy Sci..

[2] Benedet A., Manuelian C.L., Zidi A., Penasa M., De Marchi M. (2019). Invited review: β-hydroxybutyrate concentration in blood and milk and its associations with cow performance. Animal.

[3] Steeneveld W., Amuta P., van Soest F.J.S., Jorritsma R., Hogeveen H. (2020). Estimating the combined costs of clinical and subclinical ketosis in dairy cows. PLoS ONE.

[4] Gordon J.L., LeBlanc S.J., Duffield T.F. (2013). Ketosis Treatment in Lactating Dairy Cattle. Vet. Clin. N. Am. Food A.

[5] Ranaraja U., Cho K., Park M., Kim S., Lee S., Do C. (2018). Genetic parameter estimation for milk β-hydroxybutyrate and acetone in early lactation and its association with fat to protein ratio and energy balance in Korean Holstein cattle. Asian Australas. J. Anim..

[6] Pryce J.E., Gaddis K.L.P., Koeck A., Bastin C., Abdelsayed M., Gengler N., Miglior F., Heringstad B., Egger-Danner C., Stock K.F. (2016). Opportunities for genetic improvement of metabolic diseases. J. Dairy Sci..

[7] Ametaj B.N. (2017). Periparturient Diseases of Dairy Cows.

[8] Duffield T.F., Lissemore K.D., McBride B.W., Leslie K.E. (2009). Impact of hyperketonemia in early lactation dairy cows on health and production. J. Dairy Sci..

[9] van der Drift S.G., van Hulzen K.J., Teweldemedhn T.G., Jorritsma R., Nielen M., Heuven H.C. (2012). Genetic and nongenetic variation in plasma and milk β-hydroxybutyrate and milk acetone concentrations of early-lactation dairy cows. J. Dairy Sci..

[10] Wang K., Hua G., Li J., Yang Y., Zhang C., Yang L., Hu X., Scheben A., Wu Y., Gong P. (2023). Duck pan-genome reveals two transposon insertions caused bodyweight enlarging and white plumage phenotype formation during evolution. iMeta.

[11] Wang K.J., Hu H.F., Tian Y.D., Li J.Y., Scheben A., Zhang C.X., Li Y.Y., Wu J.F., Yang L., Fan X.W. (2021). The Chicken Pan-Genome Reveals Gene Content Variation and a Promoter Region Deletion in Affecting Body Size. Mol. Biol. Evol..

[12] Hu Z.L., Park C.A., Reecy J.M. (2019). Building a livestock genetic and genomic information knowledgebase through integrative developments of Animal QTLdb and CorrDB. Nucleic Acids Res..

[13] Huang H., Cao J., Hanif Q., Wang Y., Yu Y., Zhang S., Zhang Y. (2019). Genome-wide association study identifies energy metabolism genes for resistance to ketosis in Chinese Holstein cattle. Anim. Genet..

[14] Freebern E., Santos D.J.A., Fang L.Z., Jiang J.C., Gaddis K.L.P., Liu G.E., VanRaden P.M., Maltecca C., Cole J.B., Ma L. (2020). GWAS and fine-mapping of livability and six disease traits in Holstein cattle. BMC Genom..

[15] Pralle R.S., Schultz N.E., White H.M., Weigel K.A. (2020). Hyperketonemia GWAS and parity-dependent SNP associations in Holstein dairy cows intensively sampled for blood β-hydroxybutyrate concentration. Physiol. Genom..

[16] Macmillan K., Helguera I.L., Behrouzi A., Gobikrushanth M., Hoff B., Colazo M.G. (2017). Accuracy of a cow-side test for the diagnosis of hyperketonemia and hypoglycemia in lactating dairy cows. Res. Vet. Sci..

[17] Browning S.R., Browning B.L. (2007). Rapid and accurate haplotype phasing and missing-data inference for whole-genome association studies by use of localized haplotype clustering. Am. J. Hum. Genet..

[18] Purcell S., Neale B., Todd-Brown K., Thomas L., Ferreira M.A.R., Bender D., Maller J., Sklar P., de Bakker P.I.W., Daly M.J. (2007). PLINK: A tool set for whole-genome association and population-based linkage analyses. Am. J. Hum. Genet..

[19] Dunn P.K., Smyth G.K. (2018). Generalized Linear Models with Examples in R.

[20] R Core Team R: A Language and Environment for Statistical Computing. https://www.R-project.org/.

[21] Misztal I., Tsuruta S., Strabel T., Auvray B., Druet T., Lee D. BLUPF90 and related programs (BGF90). Proceedings of the 7th World Congress on Genetics Applied to Livestock Production.

[22] Aguilar I., Tsuruta S., Masuda Y., Lourenco D., Legarra A., Misztal I. BLUPF90 suite of programs for animal breeding with focus on genomics. Proceedings of the World Congress on Genetics Applied to Livestock Production.

[23] Legarra A., Aguilar I., Misztal I. (2009). A relationship matrix including full pedigree and genomic information. J. Dairy Sci..

[24] Liu X., Yin L., Zhang H., Li X., Zhao S. (2022). Performing Genome-Wide Association Studies Using rMVP. Methods Mol. Biol..

[25] Liu X., Huang M., Fan B., Buckler E.S., Zhang Z. (2016). Iterative Usage of Fixed and Random Effect Models for Powerful and Efficient Genome-Wide Association Studies. PLoS ONE.

[26] Wang Q.S., Tian F., Pan Y.C., Buckler E.S., Zhang Z.W. (2014). A SUPER Powerful Method for Genome Wide Association Study. PLoS ONE.

[27] Bland J.M., Altman D.G. (1995). Multiple significance tests: The Bonferroni method. BMJ.

[28] Bu D.C., Luo H.T., Huo P.P., Wang Z.H., Zhang S., He Z.H., Wu Y., Zhao L.H., Liu J.J., Guo J.C. (2021). KOBAS-i: Intelligent prioritization and exploratory visualization of biological functions for gene enrichment analysis. Nucleic Acids Res..

[29] Szklarczyk D., Kirsch R., Koutrouli M., Nastou K., Mehryary F., Hachilif R., Gable A.L., Fang T., Doncheva N.T., Pyysalo S. (2023). The STRING database in 2023: Protein-protein association networks and functional enrichment analyses for any sequenced genome of interest. Nucleic Acids Res..

[30] Koeck A., Jamrozik J., Schenkel F.S., Moore R.K., Lefebvre D.M., Kelton D.F., Miglior F. (2014). Genetic analysis of milk β-hydroxybutyrate and its association with fat-to-protein ratio, body condition score, clinical ketosis, and displaced abomasum in early first lactation of Canadian Holsteins. J. Dairy Sci..

[31] Benedet A., Costa A., De Marchi M., Penasa M. (2020). Heritability estimates of predicted blood β-hydroxybutyrate and nonesterified fatty acids and relationships with milk traits in early-lactation Holstein cows. J. Dairy Sci..

[32] Lou W., Zhang H., Luo H., Chen Z., Shi R., Guo X., Zou Y., Liu L., Brito L.F., Guo G. (2023). Genetic analyses of blood β-hydroxybutyrate predicted from milk infrared spectra and its association with longevity and female reproductive traits in Holstein cattle. J. Dairy Sci..

[33] Oikonomou G., Valergakis G.E., Arsenos G., Roubies N., Banos G. (2008). Genetic profile of body energy and blood metabolic traits across lactation in primiparous Holstein cows. J. Dairy Sci..

[34] Aguilar I., Misztal I., Johnson D.L., Legarra A., Tsuruta S., Lawlor T.J. (2010). A unified approach to utilize phenotypic, full pedigree, and genomic information for genetic evaluation of Holstein final score. J. Dairy Sci..

[35] Oliveira H.R., Lourenco D.A.L., Masuda Y., Misztal I., Tsuruta S., Jamrozik J., Brito L.F., Silva F.F., Schenkel F.S. (2019). Application of single-step genomic evaluation using multiple-trait random regression test-day models in dairy cattle. J. Dairy Sci..

[36] Vukasinovic N., Bacciu N., Przybyla C.A., Boddhireddy P., DeNise S.K. (2017). Development of genetic and genomic evaluation for wellness traits in US Holstein cows. J. Dairy Sci..

[37] Gaddis K.L.P., Cole J.B., Clay J.S., Maltecca C. (2014). Genomic selection for producer-recorded health event data in US dairy cattle. J. Dairy Sci..

[38] Ziyatdinov A., Kim J., Prokopenko D., Privé F., Laporte F., Loh P.R., Kraft P., Aschard H. (2021). Estimating the effective sample size in association studies of quantitative traits. G3 Genes Genom. Genet..

[39] Spencer C.C.A., Su Z., Donnelly P., Marchini J. (2009). Designing Genome-Wide Association Studies: Sample Size, Power, Imputation, and the Choice of Genotyping Chip. PLoS Genet..

[40] Soares R.A.N., Vargas G., Duffield T., Schenkel F., Squires E.J. (2021). Genome-wide association study and functional analyses for clinical and subclinical ketosis in Holstein cattle. J. Dairy Sci..

[41] Klein S.L., Scheper C., May K., König S. (2020). Genetic and nongenetic profiling of milk β-hydroxybutyrate and acetone and their associations with ketosis in Holstein cows. J. Dairy Sci..

[42] Gomez-Lopera N., Alfaro J.M., Leal S.M., Pineda-Trujillo N. (2019). Type 1 diabetes loci display a variety of native American and African ancestries in diseased individuals from Northwest Colombia. World J. Diabetes.

[43] Li C., Wang M., Zhang T.Y., He Q.Y., Shi H.P., Luo J., Loor J.J. (2019). Insulin-induced gene 1 and 2 isoforms synergistically regulate triacylglycerol accumulation, lipid droplet formation, and lipogenic gene expression in goat mammary epithelial cells. J. Dairy Sci..

[44] Lisowski P., Kosciuczuk E.M., Goscik J., Pierzchala M., Rowinska B., Zwierzchowski L. (2014). Hepatic transcriptome profiling identifies differences in expression of genes associated with changes in metabolism and postnatal growth between Hereford and Holstein-Friesian bulls. Anim. Genet..

[45] Weber C., Schäff C.T., Kautzsch U., Börner S., Erdmann S., Görs S., Röntgen M., Sauerwein H., Bruckmaier R.M., Metges C.C. (2016). Insulin-dependent glucose metabolism in dairy cows with variable fat mobilization around calving. J. Dairy Sci..

[46] Gross J., van Dorland H.A., Schwarz F.J., Bruckmaier R.M. (2011). Endocrine changes and liver mRNA abundance of somatotropic axis and insulin system constituents during negative energy balance at different stages of lactation in dairy cows. J. Dairy Sci..

[47] Kerestes M., Faigl V., Kulcsár A., Balogh O., Földi J., Fébel H., Chilliard Y., Huszenicza G. (2009). Periparturient insulin secretion and whole-body insulin responsiveness in dairy cows showing various forms of ketone pattern with or without puerperal metritis. Domest. Anim. Endocrinol..

[48] Gaddis K.L.P., Megonigal J.H., Clay J.S., Wolfe C.W. (2018). Genome-wide association study for ketosis in US Jerseys using producer-recorded data. J. Dairy Sci..

[49] Tufarelli V., Puvača N., Glamočić D., Pugliese G., Colonna M.A. (2024). The Most Important Metabolic Diseases in Dairy Cattle during the Transition Period. Animals.

[50] Ha S., Kang S., Jeong M., Han M., Lee J., Chung H., Park J. (2023). Characteristics of Holstein cows predisposed to ketosis during the post-partum transition period. Vet. Med. Sci..

[51] Pan A., Sun X.M., Huang F.Q., Liu J.F., Cai Y.Y., Wu X., Alolga R.N., Li P., Liu B.L., Liu Q. (2022). The mitochondrial β-oxidation enzyme HADHA restrains hepatic glucagon response by promoting β-hydroxybutyrate production. Nat. Commun..

[52] Xu L.N., Shi L.J., Liu L., Liang R.B., Li Q., Li J.G., Han B., Sun D.X. (2019). Analysis of Liver Proteome and Identification of Critical Proteins Affecting Milk Fat, Protein, and Lactose Metabolism in Dariy Cattle with iTRAQ. Proteomics.

[53] Tam V., Patel N., Turcotte M., Bossé Y., Paré G., Meyre D. (2019). Benefits and limitations of genome-wide association studies. Nat. Rev. Genet..

[54] McCarthy M.I., Abecasis G.R., Cardon L.R., Goldstein D.B., Little J., Ioannidis J.P.A., Hirschhorn J.N. (2008). Genome-wide association studies for complex traits: Consensus, uncertainty and challenges. Nat. Rev. Genet..

