# Supplementary material for: Genetic Background of Blood β-Hydroxybutyrate Acid Concentrations in Early-Lactating Holstein Dairy Cows Based on Genome-Wide Association Analyses
Source: Genes (Basel). 2024 Mar 26;15(4):412. doi: 10.3390/genes15040412 (PMC11049649; doi:10.3390/genes15040412)
Supplement: Supplementary file 1 [file genes-15-00412-s001.zip › genes-2924421-supplementary.pdf]

Supplementary Table S1. Descriptive statistics of blood BHB trait for individuals with genotypes in different parity.

Parity[1]	num	Mean	SD	Min	Max
BHB_2	844	0.792	0.437	0.100	5.000
BHB_3	695	0.876	0.461	0.100	3.900
$aBHB_{II}$[4]	1030	-0.087	0.099	-0.310	0.594

[1] represents the number of individuals with both genotype and phenotype values; [2] BHB_2 and BHB_3 represent phenotypic values for blood BHB; [4] $aBHB_{II}$ represents DMU-estimated breeding values for BHB.

Supplementary Table S2. The Kyoto Encyclopedia of Genes and Genomes (KEGG) pathways and Gene Ontology (GO)involved in candidate genes(*P*<0.05).

Term	Database	ID	*P*-Value	Input
Fatty acid elongation	KEGG PATHWAY	bta00062	1.16×10^{-3}	*HADHB, HADHA*
beta-Alanine metabolism	KEGG PATHWAY	bta00410	1.74×10^{-3}	*HADHA, SMOX*
Fatty acid degradation	KEGG PATHWAY	bta00071	2.33×10^{-3}	*HADHB, HADHA*
Valine, leucine and isoleucine degradation	KEGG PATHWAY	bta00280	3.37×10^{-3}	*HADHB, HADHA*
Fatty acid metabolism	KEGG PATHWAY	bta01212	4.29×10^{-3}	*HADHB, HADHA*
Metabolic pathways	KEGG PATHWAY	bta01100	9.21×10^{-3}	*PANK2, SMOX, HADHB, HADHA, NDST4, SELENOI, DNMT3A*
Progesterone-mediated oocyte maturation	KEGG PATHWAY	bta04914	9.42×10^{-3}	*ANAPC1, CDC25B*
MicroRNAs in cancer	KEGG PATHWAY	bta05206	9.88×10^{-3}	*CDC25B, DNMT3A, BCL2L11*
Phosphonate and phosphinate metabolism	KEGG PATHWAY	bta00440	1.13×10^{-2}	*SELENOI*
Cell cycle	KEGG PATHWAY	bta04110	1.76×10^{-2}	*ANAPC1, CDC25B*
Pantothenate and CoA biosynthesis	KEGG PATHWAY	bta00770	3.20×10^{-2}	*PANK2*
Transcriptional misregulation in cancer	KEGG PATHWAY	bta05202	3.81×10^{-2}	*RUNX2, SUPT3H*

Glycosaminoglycan biosynthesis-heparan sulfate/heparin	KEGG PATHWAY	bta00534	3.98×10^{-2}	*NDST4*
Butanoate metabolism	KEGG PATHWAY	bta00650	4.60×10^{-2}	*HADHA*
chromatin binding	Gene Ontology	GO:0003682	2.66×10^{-4}	*RUNX2, ASXL2, SATB2, DNMT3A, CENPB*
osteoblast development	Gene Ontology	GO:0002076	3.45×10^{-4}	*RUNX2, SATB2*
histone acetyltransferase activity	Gene Ontology	GO:0004402	9.47×10^{-4}	*PHF10, SUPT3H*
T cell homeostasis	Gene Ontology	GO:0043029	9.47×10^{-4}	*ZC3H8, BCL2L11*
positive regulation of mitotic cell cycle	Gene Ontology	GO:0045931	1.02×10^{-3}	*CDC25B, SMOC2*
odontogenesis of dentin-containing tooth	Gene Ontology	GO:0042475	2.23×10^{-3}	*RUNX2, BCL2L11*
odontogenesis of dentin-containing tooth	Gene Ontology	GO:0042475	2.23×10^{-3}	*RUNX2, BCL2L11*
endosomal transport	Gene Ontology	GO:0016197	2.44×10^{-3}	*AP5S1, RAB10*
microtubule binding	Gene Ontology	GO:0008017	3.69×10^{-3}	*SPEF1, BCL2L11, KIF3C*
cellular response to insulin stimulus	Gene Ontology	GO:0032869	3.88×10^{-3}	*RAB10, INSIG2*
roof of mouth development	Gene Ontology	GO:0060021	4.02×10^{-3}	*INSIG2, SATB2*
spermatogenesis	Gene Ontology	GO:0007283	4.26×10^{-3}	*DNMT3A, BCL2L11, MERTK*
